# A case report of transmission and disease caused by *Mycobacterium caprae* and *Mycobacterium bovis* in Lima, Peru

**DOI:** 10.1186/s12879-021-06944-5

**Published:** 2021-12-20

**Authors:** Amber Shrestha, Janeth Picoy, Arturo Torres, David A. Moore, Robert H. Gilman, Jorge Coronel, Louis Grandjean

**Affiliations:** 1 Department of Infectious Disease, Great Ormond Street Hospital for Children, London, WC1N 3JH UK; 2 Department of Infectious Disease, Diresa Callao Jr, Colina #879, Bellavista, 07016 Lima, Peru; 3 Department of Infectious Disease, Imperial College London, South Kensington, London, SW7 2AZ UK; 4 TB Centre, London School of Hygiene & Tropical Medicine, London, WC1E 7HT UK; 5 Laboratorio de Tuberculosis, Laboratorios de Investigación Y Desarrollo, Facultad de Ciencias Y Filosofía, Universidad Peruana Cayetano Heredia, Lima, Peru; 6 Department of International Health, School of Public Health, Johns Hopkins University, Baltimore, MD USA; 7 Laboratorio de Investigación de Enfermedades Infecciosas, Universidad Peruana Cayetano Heredia: Lima, Lima, Peru; 8 University College London, Gower St, Bloomsbury, London, WC1E 6BT UK

**Keywords:** Mycobacterium tuberculosis, Zoonotic disease, Caprae, Bovis, Case report

## Abstract

**Background:**

The Tuberculosis (TB) burden in Peru is significant with respect to both disease morbidity and mortality. Furthermore the recent diversification of farming enterprise to include a wide range of animal species has necessitated the consideration of members of the Mycobacterium Tuberculosis Complex (MTBC) with the potential for zoonotic transmission. *M. bovis* and *M. caprae*, a lesser known member of the MTBC exhibit an exceptionally wide host spectrum in animals and are capable of causing disease in humans. *M. bovis* has a predictable resistance profile which includes resistance to pyrazinamide. Thus, failure to identify *M. bovis* as the causative agent in reported TB cases leads to higher levels of treatment failure and contributes to the transmission of drug-resistant TB.

**Case presentation:**

Reported here are the clinical presentations, investigations and treatment histories of two patients identified from a population level genotyping study in Lima, Peru that were at the time of treatment thought to be *M. tuberculosis* patients but in retrospect were spectated using whole genome sequencing as *M. caprae* and *M. Bovis.*

**Conclusions:**

The cases reported here constitute convincing evidence that *M. caprae* and *M. bovis* are causative agents of TB infection in humans in Peru and underscore the importance of species-level MTBC member identification to effectively control and treat zoonotic TB. Furthermore these cases highlight the challenges of using clinical risk factors to identify cases of zoonotic TB in humans as their clinical presentation and transmission history is often difficult to distinguish from anthroponotic TB.

## Background

In 2018 Tuberculosis (TB) claimed 1.5 million lives and was responsible for more deaths annually than HIV and Malaria combined making it the world’s most deadly infectious disease [1]. Peru was the second biggest contributor to the TB disease burden in the World Health Organisation (WHO) region of the Americas, accounting for 13% of the 289,000 new and relapse TB cases reported in 2018 [1]. This translates to an annual incidence of 123 cases per 100,000. These figures remind us that TB is a significant threat to the population of Peru and underscores the importance of continued efforts to target and eradicate TB worldwide [1].

The aetiological agents of TB are members of the Mycobacterium Tuberculosis Complex (MTBC); namely *M. tuberculosis, M. africanum, M. bovis, M. microti, M. canetti, M. caprae and M. pinnipedii* [2]*.* The zoonotic potential of the MTBC is well established. This is perhaps best exemplified by *M. bovis* which exhibits an exceptionally wide host spectrum [2–4]. While *M. bovis* is classically associated with disease in cattle, it has also demonstrated its pathogenicity across a wide range of domestic and wild animals including but not limited to cats, dogs, badgers, bison, deer, goats and sheep [2]. Furthermore the zoonotic transmission of *M. bovis* is well documented and occurs primarily through the consumption of unpasteurised animal products contaminated with mycobacteria and through the inhalation of infectious aerosols during the close contact of humans with infected cattle. The respiratory route of human-to-human contagion is thought to be less likely for *M. bovis* relative to *M. tuberculosis* but this is disputed [5–8].

*M. bovis* exhibits an intrinsic resistance to pyrazinamide, a first-line anti-TB agent that is included in the standard treatment regimen offered to new TB patients [8–10]. Furthermore, reports of primary resistance to isoniazid, rifampicin and streptomycin primary resistance have recently emerged [3, 8]. Multidrug resistant (MDR) forms of *M. bovis* have also been isolated [8].

**Fig. 1:**
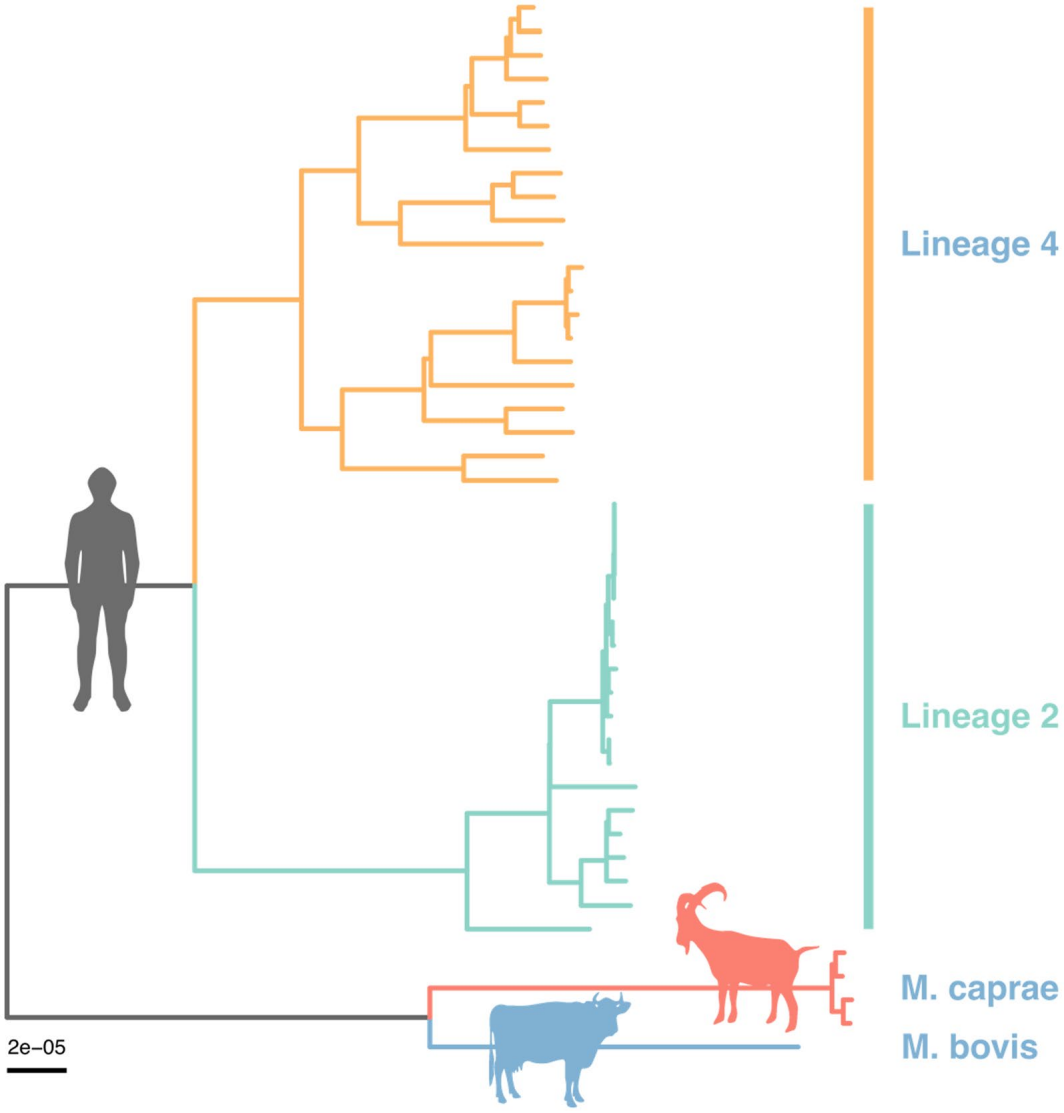
Phylogeny of *M. tuberculosis, M. bovis and M. caprae*. Shown in the figure is the maximum likelihood tree of *M. tuberculosis*, *M. bovis* and *M. caprae* based on the single nucleotide polymorphism alignment from Illumina whole genome sequencing of 2,139 clinical isolates isolates in Lima Peru

The recent diversification of farming enterprise to include animal species other than cattle has necessitated the consideration of other aetiological agents of zoonotic TB including the opportunistic human pathogen, *M. caprae*. Figure 1 shows the phylogeny of *M. caprae, M. bovis* and their genetic distance from *M. tuberculosis*. In contrast to *M. bovis,* resistance of *M. caprae* against first-line anti-TB agents is a rarity [11]. Additionally *M. caprae* exhibits a more restricted host-range relative to *M. bovis*, its primary animal reservoir is small ruminants such as sheep and goat [12]. Finally *M. caprae* is not globally distributed and rarely occurs due to strain importation. The species is thought to be confined to Europe [13, 14].

Here we present the treatment history and clinical outcomes of two patients identified from a population level genotyping study in Peru that were at the time of treatment thought to be *M. tuberculosis* patients but in retrospect were speciated using whole genome sequencing as *M. caprae* and *M. bovis.*

## Methods

### Data collection

Sample collection was undertaken as per Grandjean et al., [15]. A total of 2139 patient with symptoms of TB presented to health posts in the regions of Callao and Lima South between December 2008 and January 2010. Sputum culture, microscopic observation drug susceptibility assay (MODS), serial smear microscopy, DNA extraction, Mycobacterial Interspersed Repetitive Units (MIRU) typing, Spoligotyping and whole genome sequencing were performed as previously described [15–18]. The dataset included three *M. caprae* isolates and one *M. bovis* isolate. Data pertaining to one M. bovis and one M. caprae case was collected by chart review. The charts of the remaining M. bovis and M. caprae cases could not be located and as a result these cases were excluded from the current case review.

### Ethical approval

Ethical approval was obtained from the Institutional Review Board of the Universidad Peruana Cayetano Heredia as part of a previously published population level study (IRB00001014) (Approval Number 57492) [15–17]. Institutional approval was obtained from the Peruvian Ministry of Health and the specific regional tuberculosis control programmes in the Callao and Lima South regions of Lima, Peru. Informed written consent was obtained from all study participants.

## Case presentation

### Case 1.* M. caprae*

In 2009, a 29-year-old Peruvian male presented with a 3-month history of a chronic cough productive of yellow/green coloured sputum with occasional haemoptysis, associated with significant fatigue, diminished appetite, weight loss (7 kg in two months), night sweats and back pain.

A chest X-ray demonstrated right-sided apical cavitation with prominent bilateral hilar lymphadenopathy. A sputum specimen was positive for acid fast bacilli. Microscopic Observed Drug Susceptibility (MODS) testing did not indicate drug resistance.

The patient was treated with a 4-drug (rifampicin, isoniazid, pyrazinamide and ethambutol) anti-TB antibiotic regimen for a course of two months. Rifampicin and isoniazid was continued for a further four months. Sputum smears became negative after one month of treatment suggestive of a favourable disease progression and six subsequent sputum smear samples were negative. A repeat chest radiograph demonstrated right-sided apical fibrous reticular infiltrates consistent with treated inactive TB. The patient was considered to be in remission and remained asymptomatic thereafter.

The patient was born in the Callao region of Peru (population size 800,000). The patient shared one bedroom with his wife, son and parents in law. The patient’s locality is known for a pig farm that employs many of the region’s residents. Our patient’s bother worked on this pig fam and had regular contact with our patient. 1-month prior to our patient’s presentation the patient’s brother was successfully treated for TB, the causative MTBC agent was not identified. The patient did not report any other contacts with domestic or wild animals and denies ingesting unpasteurised dairy products. There was no further household transmission of TB between our patient and the other members of his household.

The patient’s sputum sample was processed on both liquid (MODS) and solid Ogawa medium. An aliquot was sub-cultured and underwent Spoligotyping after DNA extraction at the Universidad Peruana Cayetano Heredia (Lime, Peru) [15, 19, 20]. The isolate was identified as *M. caprae* and was further genotyped using a 15-loci MIRU-VNTR analysis at the Kobe Institute (Kobe, Japan) following established protocols [21]. Subsequent whole genome sequencing identified the sample as *M. caprae*.

### Case 2.* M. bovis*

In 2008, a 64-year-old Peruvian male presented with a 3-month history of a productive cough with haemoptysis and shortness of breath. A sputum smear was positive for acid fast bacilli. MODS testing did not indicate drug resistance. A chest radiograph demonstrated apical cavitation of the left upper and middle lung lobe with blunting of the left costo-diaphagmatic angle.

The patient was treated with the standard 4-drug regimen for a total course of 6 months. Sputum smears became negative after one month of treatment suggested favourable disease progression and five subsequent sputum smears were negative.

However, 1 month after the patient stopped treatment he deteriorated clinically and had three positive smears. He was treated with a second line anti-TB regimen of ethambutol, pyrazinamide, ethionamide, ciprofloxacin, cycloserine, kanamycin and para-aminosalicylic acid for a total duration of 18 months. Despite initial improvements in symptoms, the patient relapsed again on 2nd line therapy and died of respiratory failure in 2012.

The patient lived alone in the region of Lima South (population size 1,200,000). Prior to his initial hospitalisation the patient had spent two months visiting family in the city of Huánuco in central Peru. This region has the greatest density of cattle farms and grazing cows in the country. While the consumption of unpasteurised milk in Huánuco is commonplace, our patient denies consuming unpasteurised dairy products.

The patient’s sputum sample was processed on both liquid (MODS) and solid Ogawa medium. An aliquot was sub-cultured and underwent Spoligotyping after DNA extraction at the Universidad Peruana Cayetano Heredia (Lime, Peru) [15, 19, 20]. The isolate was identified as *M. bovis* and was further genotyped using a 15-loci MIRU-VNTR analysis at the Kobe Institute (Kobe, Japan) following established protocols [21]. Subsequent whole genome sequencing identified the sample as *M. bovis*.

## Discussion and conclusion

The cases reported here constitute convincing evidence that *M. caprae* and *M. bovis* are causative agents of TB infection in humans in Peru. Furthermore these cases highlight the challenges of using clinical risk factors to identify cases of zoonotic TB in humans as their clinical presentation and transmission history is often difficult to distinguish from anthroponotic TB.

In Peru small ruminant farming is a meaningful source of enterprise and sustenance by providing meat, milk and wool to Peruvian communities who’s semi-arid and arid land often lacks the rich pasture necessary for cattle farming. More recently small ruminants such as the domestic caprid, the alpaca and the llama have demonstrated their ability to generate revenue in Peru’s growing tourism industry and this has further bolstered their value. As the primary animal reservoir for *M. caprae*, it could be assumed that Peru’s large inventory of small ruminants would result in a large proportion of reported TB cases being attributed to *M. caprae*. However *M. caprae* accounts for a mere 0.3% of human TB cases with the great majority of these cases being reported in Europe [14]. In fact, to the best of our knowledge there are only two documented cases of *M. Caprae* infection in humans outside of Europe; both described in Morocco [22]. Similarly, *M. caprae* is rarely isolated from animals outside of Europe. Isolates from cattle have been described in Tunisia and Algeria only, while the only documentation of *M. caprae* in China comes from Zeng et al., who described its isolation from one reindeer and one sheep [13, 23]. Thus, the case presented here represents the first documentation of zoonotic *M. caprae* infection in humans in The Americas, and only the fourth documented isolation outside of continental Europe.

It is unlikely that the apparent scarcity of *M. caprae* outside of Europe reflects the true epidemiology of zoonotic *M. caprae* in Peru but rather the lack of differentiation between *M. caprae* and the other members of the MTBC as the offending pathogen in reported TB cases [11, 13]. *M. caprae* and *M. tuberculosis* have a very similar disease evolution and they are both typically drug-susceptible [14]. It is interesting to note that while *M. caprae* and *M. bovis* are often associated with extra-pulmonary presentations of TB, both of our patients presented with a symptoms profile consistent with classical pulmonary TB [13, 14, 24]. Additionally when asked as part of a TB-specific history taking proforma included in each of the patients charts, neither of our patients reported consuming unpasteurised dairy products and both denied direct contact with cattle or caprine herds. This highlights the similarities in clinical picture between TB caused by M. bovis or M. caprae and TB infection caused by M. tuberculosis. Furthermore this would suggest that the screening questions addressing the risk factors of zoonotic TB, namely questions pertaining to animal product consumption and/or contact, are not as sensitive as one would have thought.

In developed countries ‘whole herd test and slaughter’ campaigns have significantly reduced but not eradicated the public health risk of zoonotic TB [25]. Herd eradication programmes aimed at reducing the zoonotic TB burden in Peru are scarce and are implemented on a regional basis relying on the active participation of farmers. These programmes have struggled to target the last vestiges of bovine TB which likely persist because of the failure of such programmes to include animal populations other than domestic cattle [25]. *M. caprae’s* existence in caprine herds, which often coinhabit with cattle herds, represents a significant pool of zoonotic TB capable of causing disease in humans that is not targeted by herd eradication programmes. Describing the true disease burden of M. caprae in Peru would allow policy makers to decide whether caprine herds’ contribution to the zoonotic TB pool in Peru warrants their inclusion in regional eradication programmes.

The rationale for the upfront species-level identification of *M. bovis* is clear. M. bovis has a predictable resistance profile which includes but is not limited pyrazinamide resistance. Thus, failure to identify M. bovis as the causative agent in reported TB cases has the potential to lead to higher levels of treatment failure and contributes to the transmission of drug-resistant TB. This was exemplified by our patient, *Case 2*. whose infection failed to respond to the first line drug regimen which included Pyrazinamide. In 2019 the WHO named Peru as one of the top 30 countries with the highest burden of MDR-TB [1]. M. Bovis is a known causative agent of MDR-TB and characterizing its epidemiology in Peru has important therapeutic implications.

Limitations of this study include the use of chart review as the primary method of data collection. It was not possible to contact our patients directly and this limited the quantity and quality of the data presented here. Furthermore, it was not possible to gather data from two of the M. caprae cases isolated as part of our parent study and this limits our ability to generalize the findings discussed here.

Recent large-scale genotyping studies have provided a population level view of circulating strains of TB in the region of Lima, Peru. As a result of this, the prevalence of human disease due to the different members of the MTBC has been quantified [16]. The case reports presented here underscore the importance of species-level MTBC member identification in efforts to control and effectively treat zoonotic TB caused by M. bovis and M. caprae.

## Data Availability

Figure 1. titled, Phylogeny of *M. tuberculosis, M. bovis and M. caprae* depicts the maximum likelihood tree of *M. tuberculosis*, *M. bovis* and *M. caprae* based on the single nucleotide polymorphism alignment from Illumina whole genome sequencing of 2,139 clinical isolates isolates in Lima Peru. This figure is derived from 2,139 clinical isolates collected as part of the following paper: Grandjean L, Iwamoto T, Lithgow A, Gilman RH, Arikawa K, Nakanishi N, Martin L, Castillo E, Alarcon V, Coronel J, Solano W, Aminian M, Guezala C, Rastogi N, Couvin D, Sheen P, Zimic M, Moore DA. The Association between Mycobacterium Tuberculosis Genotype and Drug Resistance in Peru. PLoS One. 2015 May 18;10(5):e0126271. https://doi.org/10.1371/journal.pone.0126271. PMID: 25,984,723; PMCID: PMC4435908. The original data set is available to request from the original authors of that paper.

## Abbreviations

*M. bovis*: *Mycobacterium tuberculosis*
*M. caprae*: *Mycobacterium caprae*
*M. Tuberculosis*: *Mycobacterium bovis*
MTBC: Mycobacterium tuberculosis complex
TB: Tuberculosis
WHO: World Health Organisation
MDR-TB: Multi-drug resistant tuberculosis
15 Locus MIRU-VNTR: 15-Locus Mycobacterial Interspersed Repetitive Unit Variable Number Tandem Repeat
MODS: Microscopic Observation Drug Susceptibility Assay
